# Cytotoxic, Antioxidant, and Enzyme Inhibitory Properties of the Traditional Medicinal Plant *Matthiola incana* (L.) R. Br.

**DOI:** 10.3390/biology9070163

**Published:** 2020-07-13

**Authors:** Maria Fernanda Taviano, Natalizia Miceli, Rosaria Acquaviva, Giuseppe Antonio Malfa, Salvatore Ragusa, Deborah Giordano, Guillermo Cásedas, Francisco Les, Víctor López

**Affiliations:** 1 Department of Chemical, Biological, Pharmaceutical and Environmental Sciences, Polo Annunziata, University of Messina, 98168 Messina, Italy; mtaviano@unime.it (M.F.T.); nmiceli@unime.it (N.M.); deboragiordano94@gmail.com (D.G.); 2 Department of Drug Science, Biochemistry Section, University of Catania, 95123 Catania, Italy; racquav@unict.it (R.A.); g.malfa@unict.it (G.A.M.); 3 Department of Health Sciences, University Magna Graecia of Catanzaro, 88100 Catanzaro, Italy; sragusa@unicz.it; 4 Department of Pharmacy, Faculty of Health Sciences, Universidad San Jorge, 50830 Villanueva de Gállego (Zaragoza), Spain; gcasedas@usj.es (G.C.); fles@usj.es (F.L.); 5 Instituto Agroalimentario de Aragón-IA2, CITA-Universidad de Zaragoza, 50013 Zaragoza, Spain

**Keywords:** anti-glucosidase, antioxidant, Brassicaceae, medicinal plants, enzyme inhibitor, polyphenols, stock flower

## Abstract

*Matthiola incana* (L.) R. Br. (Brassicaceae) is widely cultivated for ornamental purposes and utilized as a medicinal plant. In the present work, the hydroalcoholic extract from the aerial parts of this species has been evaluated in different bioassays in order to detect potential pharmacological applications. The cytotoxic capacity against the human colorectal adenocarcinoma (CaCo-2) and breast cancer (MCF-7) cell lines was tested using the MTT (3-(4,5-dimethylthiazol-2-yl)-2,5-diphenyltetrazolium bromide) assay. The extract was investigated as a neuroprotective inhibitor of central nervous system (CNS) enzymes such as monoamine oxidase A, tyrosinase, acetylcholinesterase, and as a natural enzyme inhibitor of α-glucosidase and lipase involved in some metabolic disorders such as obesity or type 2 diabetes. The antioxidant ability was also evaluated in an enzymatic system (xanthine/xanthine oxidase assay). Results showed that the *M. incana* extract displayed moderate to low cytotoxicity vs. CaCo-2 cells. The extract acted as a superoxide radical scavenger and enzymatic inhibitor of monoamine oxidase A, tyrosinase, α-glucosidase, and lipase. The best results were found in the α-glucosidase assay, as *M. incana* hydroalcoholic extract was able to inhibit the enzyme α-glucosidase up to 100% without significant differences, compared to the antidiabetic drug acarbose. *Matthiola incana* has been demonstrated to exert different biological properties. These are important in order to consider this species as a source of bioactive compounds.

## 1. Introduction

*Matthiola incana* (L.) R. Br. (Brassicaceae), usually called “stock flower”, is widely cultivated for ornamental purposes in many regions around the world [1,2].

This species is utilized in the traditional medicine of several countries (Iran, India, Ecuador, Bolivia, Italy) for the treatment of various ailments, including inflammations and cancer, particularly breast and testicular cancer [3,4,5,6,7,8,9,10,11].

*Matthiola incana* is also utilized as an edible plant; the flowers are eaten as a vegetable or used as a garnish, especially with sweet desserts [7,12], and the freshly boiled pods are consumed in Italy (Puglia). In China, the edible flowers are sold in health food stores and consumed in the form of tea [13,14].

Considering all the above-mentioned uses of *M. incana*, our research team recently published a study reporting the phytochemical composition and the biological properties of a hydroalcoholic extract (80% methanol) obtained from the aerial parts (leaves and flower buds) of *M. incana* growing wild in Sicily, Italy. In our previous work, the phenolic compounds and volatile constituents were characterized for the first time. The extract was found to possess antioxidant activity in both in vitro and in vivo assays. In particular, it showed good chelating properties and a protective effect on *Escherichia coli* growth and survival from the oxidative stress induced by hydrogen peroxide. Moreover, the extract proved non-toxic against brine shrimp larvae (*Artemia salina* Leach), indicating its potential safety [15].

As part of the ongoing research, the present work was designed to assess further biological activities of the same extract using a wide variety of in vitro bioassays.

Starting from its traditional use in the prevention and treatment of different types of cancers, including breast cancer, the cytotoxicity of the extract against two different human cancer cell lines, breast (MCF-7) and colorectal adenocarcinoma (CaCo-2), was tested through the MTT (3-(4,5-dimethylthiazol-2-yl)-2,5-diphenyltetrazolium bromide) assay in order to provide a scientific basis for the empiric use of *M. incana*.

In recent years, ever-growing evidence indicates that oxidative stress plays a key role in the pathogenesis of a number of diseases associated with neurodegeneration and of many metabolic disorders [16,17]. Further, increasing evidence suggests a link between the incidence and progression of some neurodegenerative disorders and metabolic dysfunction [18]. Antioxidants can provide the desired antioxidant status, therefore contributing to the prevention of these pathologies; for this reason, oxidative stress is a therapeutic target. Indeed, several research studies have addressed the ability of natural antioxidants to delay or prevent neurodegenerative and metabolic disorders.

Taking into account the antioxidant activity of *M. incana* extract previously demonstrated, the second aim of this work is to assay the antioxidant capacity of the extract in an enzymatic system and to investigate its effect as a neuroprotective inhibitor of central nervous system (CNS) enzymes and as an enzyme inhibitor of α-glucosidase and lipase.

The ability to modulate key physiological enzymes has been investigated using monoamine oxidase A (MAO-A), tyrosinase (TYR), acetylcholinesterase (AChE), whose inhibition may lead to a neuroprotective effect [19,20,21], and lipase and α-glucosidase (α-GLU), to establish the extract’s anti-obesity and antidiabetic potential [22].

## 2. Materials and Methods

### 2.1. Reagents and Chemicals

Xanthine, nitroblue tetrazolium (NBT), xanthine oxidase, gallic acid, galantamine, acetylthiocholine iodide (ATCI), 5,5’-dithiobis-(2-nitrobenzoic acid) (DTNB), tris, acetylcholinesterase from electric eel (AChE), vanillic acid, 4-aminoantipyrine, horseradish peroxidase, tyramine, monoamine oxidase A (MAO-A), levodopa (L-DOPA), tyrosinase (TYR), orlistat, lipase (type II) from porcine pancreas, p-nitrophenyl butyrate (pNPB), α-glucosidase (α-GLU) from *Saccharomyces cerevisiae,* and p-nitrophenyl glucopyranoside (pNPG) were acquired through Sigma-Aldrich (Madrid, Spain); clorgyline, kojic acid, and acarbose were from Cymit Quimica (Barcelona, Spain); Na_2_CO_3_, HCl, NaCl, MgCl_2_, CaCl_2_, MeOH, and potassium phosphate were from Panreac (Barcelona, Spain), and fetal bovine serum was from Gibco (Barcelona, Spain). All standards used for comparative purposes in the bioassays and experiments were acquired with a purity of at least 99%. All the chemicals and reagents not mentioned above were purchased from Sigma-Aldrich (Milano, Italy).

### 2.2. Plant Material and Extraction

The aerial parts (leaves and flower buds) of *Matthiola incana* (L.) R. Br. were collected in March, just before flowering, around Capo d’Orlando (Messina, Sicily, Italy). The taxonomic identification was confirmed by Prof. S. Ragusa, Department of Health Sciences, University “Magna Graecia” of Catanzaro. Voucher specimens were deposited in the Herbarium of the Department of Chemical, Biological, Pharmaceutical and Environmental Sciences, University of Messina, under accession number 406/17. After harvesting, the aerial parts were frozen, then the freeze-dried plant material (25.76 g) was subjected to preventive maceration with 80% MeOH (250 mL) for 150 min. The extraction was carried out with 80% MeOH (250 mL) in an ultrasonic bath at 50 °C for 15 min (three times). The filtrates were combined and evaporated to dryness by rotavapor; the yield of the extract, referred to 100 g of lyophilized plant material, was 33.33%.

### 2.3. Cytotoxic Activity

#### 2.3.1. Cell Culture and Treatments

Human colorectal adenocarcinoma cells (CaCo-2), obtained from the American Type Culture Collection (Rockville, MD, USA), were cultured as previously described [23]. The cells were plated at a constant density of 3 × 10^5^/mL in order to obtain identical experimental conditions in the different tests and to achieve high accuracy of measurement. MCF-7 breast cancer cells (ATCC cell bank, Rockville, MD, USA) were cultured in RPMI medium containing 10% fetal bovine serum (FBS), 100 U/mL penicillin, and 100 μg/mL streptomycin in 5% CO_2_ at 37 °C and seeded at a constant density of 3 × 10^5^/mL. Both cell lines were seeded in 96-well plates for MTT assay and in 6-well plates for lactate dehydrogenase (LDH) release. At sub-confluent conditions, CaCo-2 and MCF-7 cells were treated with different concentrations of the *M. incana* extract for 48 and 72 h. The extract was dissolved in medium to obtain final concentrations ranging from 0.0625 to 4 mg/mL. After the treatments, cells were scraped, washed with PBS, and subsequently utilized for analysis.

#### 2.3.2. MTT Bioassay

The MTT assay was performed according to Malfa and collaborators [24] in order to assess cell viability. After 24 h, cells were treated with different concentrations of extract (0.0625–4 mg/mL) for 48 and 72 h. The optical density was measured with a microplate spectrophotometer reader (Titertek Multiskan, Flow Laboratories, Helsinki, Finland) at λ = 570 nm. The results were expressed as a percentage of cell viability with respect to control (untreated cells).

#### 2.3.3. LDH Release

Necrotic cell death was measured by LDH release as a consequence of cell membrane disruption. Enzyme activity was measured, in cell culture medium and in the cellular lysates, spectrophotometrically at λ = 340 nm through the reduction of oxidized nicotinamide adenine dinucleotide (NAD) [23]. LDH release was calculated as a percentage of the total amount, as a sum of the enzymatic activity in the cellular lysate and in the culture medium. The results were expressed as a percentage of LDH released.

### 2.4. Antioxidant Activity: Superoxide Radical Scavenging Activity

The xanthine/xanthine oxidase assay was used to assess the ability of *M. incana* extract to eliminate superoxide radicals generated by the reaction [25]. The assay was performed in 96-well microplates, and the mixture contained 90 μM of xanthine, 16 mM of Na_2_CO_3_, 22.8 μM of NBT (solved in phosphate buffer, pH 7), and sample or reference inhibitor (gallic acid) at different concentrations. Then, xanthine oxidase (168 U/L) was added to start the reaction. The mixture was incubated for 2 min at 37 °C. Controls were performed in order to obtain 100% activity, containing buffer instead of samples or inhibitors. Blanks were also performed, in order to avoid background interference, containing buffer instead of the enzyme. The inhibitory activity of the samples was measured by absorbance at 560 nm, as a result of the NBT transformation into the chromogen formazan formed by the superoxide radical. The results were expressed as a percentage of free radical scavenging capacity (RSC) and were calculated with Equation (1) considering blank substraction.

RSC (%) = [(Abs_control_ − Abs_sample_)/Abs_control_] × 100 (1)

The *M. incana* extract activity on the xanthine oxidase enzyme was also evaluated by measuring the formation of uric acid from xanthine at 295 nm after 2 min. The reaction mixture contained the same components as those described previously in the xanthine/xanthine oxidase system, with the exception of NBT.

### 2.5. Enzyme Inhibition Activity

#### 2.5.1. Acetylcholinesterase (AChE) Inhibition

The activity was measured using a 96-well microplate reader based on Ellman’s method with some modifications [25]. The assay mixture contained 15 mM ATCI in Millipore water, 3 mM DTNB in buffer C (50 mM Tris-HCl, pH 8, 0.1 M NaCl, 0.02 M MgCl_2_·6 H_2_O), buffer (50 mM Tris-HCl, pH 8, 0.1% fetal bovine serum), and different concentrations of *M. incana* extract or reference inhibitor solved in buffer (50 mM Tris-HCl, pH 8). Then, AChE (0.22 U/L) was added to start the reaction. Controls were performed in order to obtain 100% activity, containing buffer instead of samples or inhibitors. Blanks were also performed, in order to avoid background interference, containing buffer instead of the enzyme. Absorbance was read 13 times every 13 s at 405 nm. Galantamine was used as a reference inhibitor. The results were expressed as a percentage of inhibition to the control wells and were calculated with Equation (2).

Inhibition (%) = [(Abs_control_ − Abs_sample_)/Abs_control_] × 100 (2)

#### 2.5.2. Monoamine Oxidase A (MAO-A) Inhibition

The assay was performed in a 96-well microplate using the technique previously described [25]. Each well contained *M. incana* extract or reference inhibitor (clorgyline) at different concentrations, chromogenic solution (0.8 mM vanillic acid, 417 mM 4-aminoantipyrine, and 4 U mL^−1^ horseradish peroxidase in potassium phosphate buffer pH 7.6), 3 mM tyramine, and 8 U/mL MAO-A. Controls were performed in order to obtain 100% activity, containing buffer instead of samples or inhibitors. Blanks were also performed, in order to avoid background interference, containing buffer instead of MAO-A. The absorbance was read at 490 nm every 5 min for 30 min. The results were calculated with Equation (2).

#### 2.5.3. Tyrosinase (TYR) Inhibition

The assay was conducted in 96-well microplates using a microplate reader to measure absorbance at 475 nm using the described procedure [26]. *M. incana* extract at 10 μL or reference inhibitor (Kojic acid) at different concentrations, L-DOPA, phosphate buffer (pH 6.8), and tyrosinase were added to each well. Controls were performed in order to obtain 100% activity, containing buffer instead of samples or inhibitors. Blanks were also performed, in order to avoid background interference, containing buffer instead of the TYR. The results were calculated with Equation (2).

#### 2.5.4. Lipase Inhibition

The activity was measured in 96-well microplates using previous protocols [27]. Each well contained *M. incana* extract or reference inhibitor (orlistat) at different concentrations and 2.5 mg/mL lipase, prepared in 100 mM Tris and 5 mM CaCl_2_ buffer, pH 7.0. After 15 min preincubation, 20 μL of 10 mM pNPB solution was added to each well for another 15 min incubation at 37 °C. Controls were performed in order to obtain 100% activity, containing buffer instead of samples or inhibitors. Blanks were also performed, in order to avoid background interference, containing buffer instead of lipase. Absorbance was read at 405 nm, and orlistat was used as a reference inhibitor. The results were calculated with Equation (2).

#### 2.5.5. Inhibition of α-Glucosidase (α-GLU)

The capacity of *M. incana* extract to inhibit α-glucosidase was measured in a 96-well microplate reader based on the method previously described [27]. Each well contained a reaction mixture of *M. incana* extract or reference inhibitor (acarbose) at different concentrations and α-GLU (1.0 U mL^−1^). After preincubation for 10 min, 3.0 mM pNPG (dissolved in 20 mM phosphate buffer, pH 6.9) was added to start the reaction and incubated at 37 °C for 20 min. Then, absorbance was measured at 405 nm. Controls were performed in order to obtain 100% activity, containing buffer instead of samples or inhibitors. Blanks were also performed, in order to avoid background interference, containing buffer instead of α-GLU. The results were calculated with Equation (2).

### 2.6. Statistical Analysis

The results of cytotoxicity tests were the mean ± standard deviation (SD) of four experiments in triplicate. One-way analysis of variance (ANOVA), followed by Bonferroni’s t-test, was performed in order to estimate significant differences among groups. Data were reported as mean values ± standard deviation (SD), and differences among groups were significant at *p* < 0.001. The results of the antioxidant and enzyme inhibiting assays were obtained from at least the average of three independent experiments and were expressed as mean ± standard error (SEM). Data analyses were run with GraphPad Prism v.6 (GraphPad Software, San Diego, CA 92108, USA).

## 3. Results and Discussion

### 3.1. Cytotoxic Activity

Even though there has been significant progress in the fight against cancer, the discovery of bioactive natural products still plays an important role in the research and development of new anticancer agents to treat it effectively and with fewer adverse effects. In order to provide a scientific basis for the empiric use of *M. incana* as an anticancer agent, we tested the cytotoxic activities of hydroalcoholic extract of this species against two human cancer cell lines: MCF7 breast cancer cells and CaCo-2 colorectal adenocarcinoma cells. No change in viability was observed in MCF-7 cells treated with 0.0625-4 mg/mL of *M. incana* extract for 48 and 72 h. This result does not support the reported traditional use of *M. incana* for the treatment of breast cancer.

**Figure 1:**
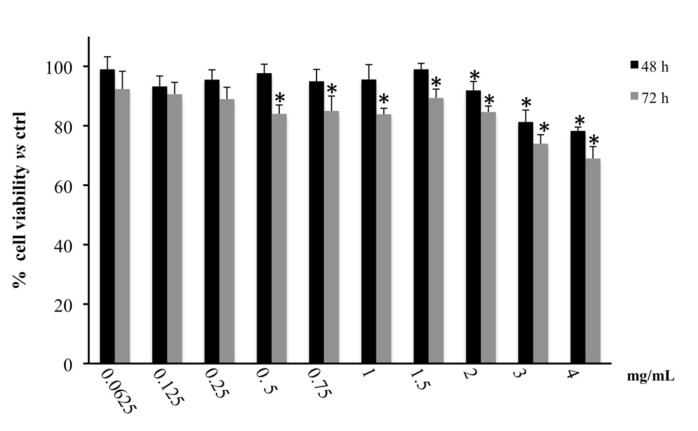
Cell viability in CaCo2 cells treated for 48 and 72 h with the hydroalcoholic extract of *M. incana* aerial parts evaluated by MTT assay. Values are the mean ± SD of four experiments in triplicate. * Significant vs. untreated control cells: *p* < 0.001.

In contrast, as shown in Figure 1, the treatment of CaCo-2 with different concentrations of *M. incana* extract induced a moderate inhibitory effect on succinate dehydrogenase activity at both 48 and 72 h of exposure. The exerted cytotoxic activity was significant, starting from the concentration of 2 mg/mL at 48 h and from the concentration of 0.5 mg/mL at 72 h of treatment, where the inhibitory effects reached a value of 30% at 4 mg/mL. This distinct effect is probably due to the two different cell lines, which respond to the same treatment in different ways; indeed, MCF7 breast cancer cells are well known for their drug resistance capacity mediated by breast cancer resistance protein (BCRP) [28].

**Figure 2:**
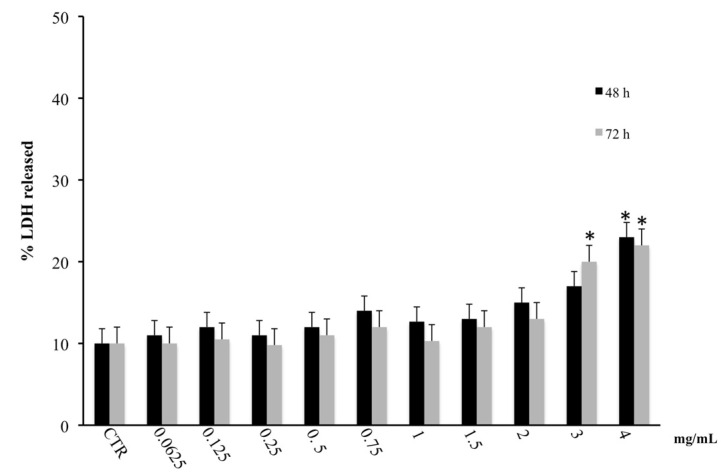
LDH released in CaCo2 cells untreated and treated for 48 and 72 h with the hydroalcoholic extract of *M. incana* aerial parts. Values are the mean ± SD of four experiments in triplicate. * Significant vs. untreated control cells: *p* < 0.001.

The cytotoxic effect detected on the CaCo-2 cell line by MTT assay was further investigated by the determination of LDH release. Figure 2 evidenced that non-necrotic cell death was associated with reduced viability at the lowest concentrations of the extract, allowing us to hypothesize the involvement of a different death pathway for both times of exposure. Conversely, at the highest concentration (4 mg/mL), we found a significant increase in LDH release suggesting a necrotic effect due to the high concentration of plant metabolites present in the extract (Figure 2).

The potential cytotoxic activity of *M. incana* extract determined in the CaCo-2 cell line could be related to its ferrous ions’ chelating properties, as demonstrated in our previous study [15]. In fact, some metals such as copper and iron have been shown to play a significant role in the rapid proliferation of cancer cells [29].

### 3.2. Antioxidant Activity

**Figure 3:**
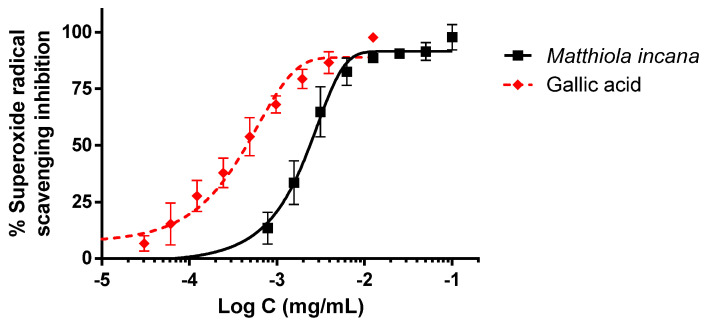
Antioxidant activity of the hydroalcoholic extract of *M. incana* aerial parts evaluated in the xanthine/xanthine oxidase system. IC_50_ values were calculated by non-linear regression. All concentrations were tested at least in triplicate, and each point represents mean ± SEM.

*Matthiola incana* extract showed the ability to scavenge superoxide radicals produced by a xanthine/xanthine oxidase reaction (Figure 3). This antioxidant activity was lower than the reference compound, gallic acid. The IC_50_ values were 2.38 and 0.45 µg/mL for *M. incana* and gallic acid, respectively.

Antioxidant properties of phenolic compounds are widely demonstrated [30]. Previous studies by the authors characterized the polyphenolic compounds contained in *M. incana* extract and showed the potential of this plant as a radical scavenger, reducing agent, or metal chelator [15]. However, this was the first time that this antioxidant potential was observed in a radical superoxide, that is, one of the physiological reactive oxygen species presented as the product of numerous enzymatic reactions.

### 3.3. Inhibitory Activities on CNS Enzymes

**Figure 4:**
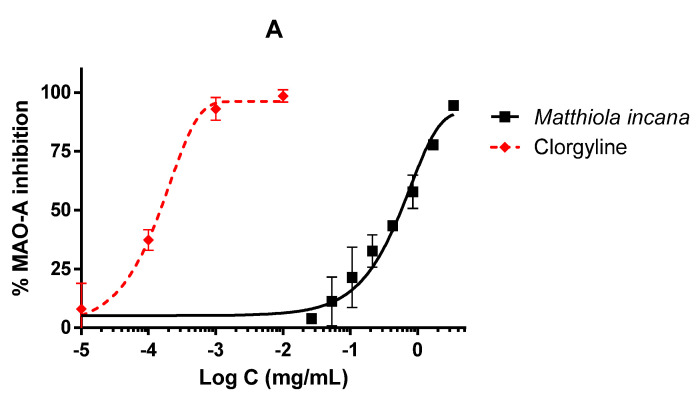

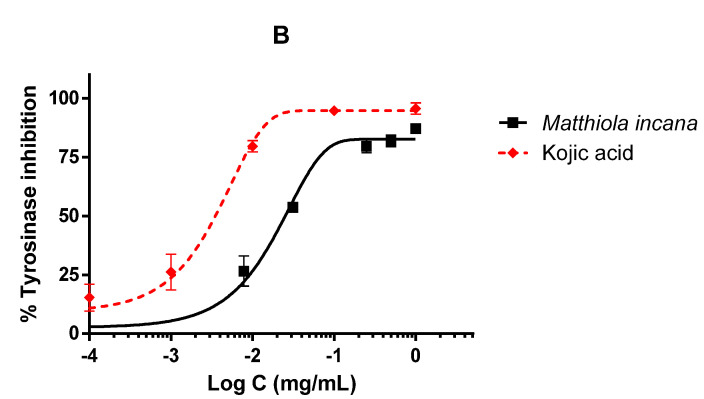
Activity of the hydroalcoholic extract of *M. incana* aerial parts in enzymes related to central nervous system (CNS) pathologies. IC_50_ values were calculated by non-linear regression. All concentrations were tested at least in triplicate, and each point represents mean ± SEM. (**A**): monoamine oxidase A (MAO-A) inhibition performed by *M. incana* extract and clorgyline as standard. (**B**): tyrosinase (TYR) inhibition by *M. incana* extract and kojic acid as standard.

*Matthiola incana* extract was able to inhibit CNS enzymes as MAO-A and TYR but not AChE. In the MAO-A inhibition assay, the extract reached complete enzyme inhibition but only at the highest doses (Figure 4A). The difference between the extract and clorgyline was obvious, as also confirmed by the IC_50_ values, 570.28 and 0.15 µg/mL for the extract and the reference inhibitor, respectively. However, the extract showed higher activity in the TYR inhibition assay, reaching almost 90% of inhibition and a very similar profile to the reference inhibitor, kojic acid (Figure 4B). The IC_50_ values were 25.21 and 3.52 µg/mL for the extract and the reference inhibitor, respectively. However, the extract was not able to inhibit AChE in any tested concentration.

This was the first time that these inhibitory properties on CNS enzymes were shown for this species. However, it is known that antioxidants and extracts with high polyphenol content could be involved in the prevention of diseases related to oxidative stress as neurodegenerative pathologies [31,32]. Synthetic MAO inhibitors have been used to treat depression or dementia but may have side effects due to the increase of biogenic amines in the blood. Kaempferol, a flavonoid contained in the extract, has already demonstrated its potential inhibitory effect on human MAO-A [33,34], which could explain the inhibition of this enzyme by the *M. incana* extract. TYR inhibition may represent a potential neuroprotective strategy preventing dopamine-induced neuronal damage, although TYR inhibitors are more often used in preventing skin pigmentation in dermatology [35]. Phenolic compounds present in this extract as kaempferol or luteolin inhibited TYR [36,37]. However, naringenin, another flavonoid contained in high concentration in the extract, exhibited significant anti-proliferative activity against B16F10 melanoma cells and enhanced TYR activity, suggesting its use as a natural tanning agent [38].

### 3.4. Inhibitory Activities on α-Glucosidase (α-GLU) and Lipase

**Figure 5:**
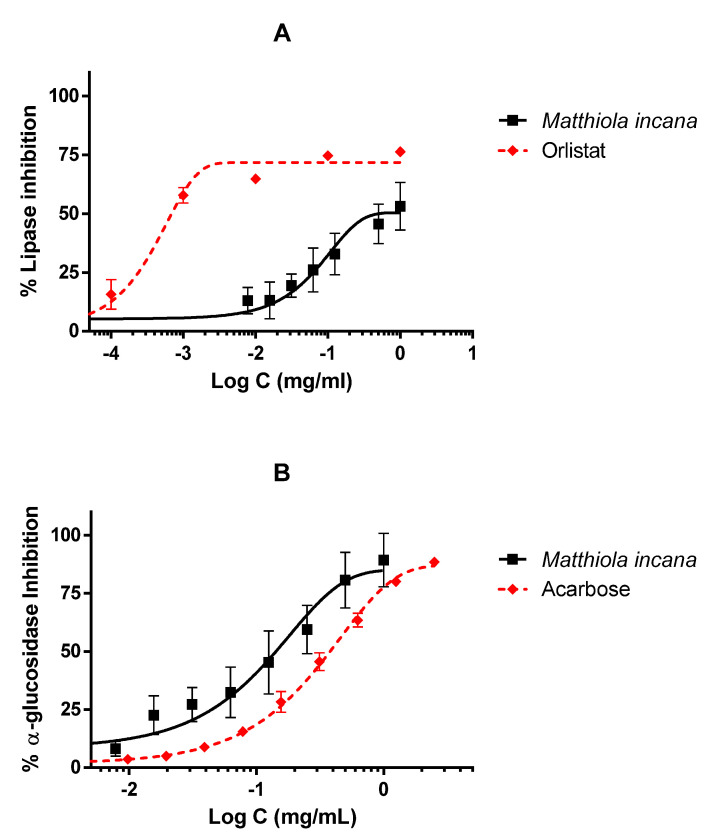
Activity of the hydroalcoholic extract of *M. incana* aerial parts on lipase (**A**) and α-glucosidase (**B**). IC_50_ values were calculated by non-linear regression. All concentrations were tested at least in triplicate, and each point represents mean ± SEM. A: Lipase inhibition performed by *M. incana* extract and orlistat as standard. B: α-glucosidase (α-GLU) inhibition by *M. incana* extract and acarbose as standard.

*Matthiola incana* extract was also tested as an inhibitor of enzymes presented in the digestive tract, with the aim of investigating the potential use of this extract as an antidiabetic and/or anti-obesity herbal drug. The extract was able to inhibit around 50% of lipase activity at the highest tested concentration (Figure 5A). IC_50_ for the extract was 508.71 µg/mL, considerably higher than reference inhibitor, orlistat, whose IC_50_ was 0.70 µg/mL. Nonetheless, the inhibitory activity of *M. incana* was better in the α-GLU assay. As shown in Figure 5B, the extract inhibited the enzyme in a dose-dependent manner with a better response than the reference inhibitor acarbose. The inhibitory curve of the extract was slightly shifted to the left relative to acarbose; however, no significant differences were detected between acarbose and the extract (142.20 and 378.92 µg/mL for the *M. incana* extract and acarbose, respectively).

This was also the first time that this extract was shown to be able to inhibit enzymes involved in metabolic disorders such as lipase and α-GLU. Although inhibitory effects were detected in commercial glucosidases from *Saccharomyces cerevisiae*, this enzyme is widely used to screen glucosidase inhibitors with potential use and applications in human health; nevertheless, more studies will be performed in the future in order to establish the inhibitory activity on human glucosidases. Oxidative stress is also responsible for metabolic syndrome pathologies [39,40], and, for this reason, different antioxidants have been studied in this field. There is only one study in which *M. incana* seeds, rich in linolenic acid oil (55–65%), reduced cholesterol levels and increased omega-3 fatty acid levels in the plasma of rats [41]. However, the phytochemical composition cannot be compared to *M. incana* aerial parts, as this extract is particularly rich in polyphenols; among flavonoids detected in this extract, kaempferol, naringenin, and luteolin were found to inhibit α-GLU [32,33,34,35,36,37,38,39,40,41,42,43,44,45,46]. Kaempferol was also able to inhibit lipase [47]. Luteolin and naringenin have been demonstrated to protect against severe acute pancreatitis in mice by exerting anti-inflammatory and antioxidant effects and decreasing lipase activity [48,49]. The ability of the isolated flavonoids presented in the extract could explain the activity of the extract in these enzymes. Moreover, another study with kaempferol demonstrated the capability to decrease adipogenesis in the 3T3-L1 cell line and lipid accumulation in mature adipocytes [50].

## 4. Conclusions

Our findings highlight that the hydroalcoholic extract of *M. incana* aerial parts displays moderate to low cytotoxicity vs. CaCo-2 cells and acts as a superoxide radical scavenger and enzymatic inhibitor of MAO-A, TYR, α-GLU, and lipase. The best results were found in the α-GLU assay, which paves the way for further studies aimed at evaluating the inhibitory effect against mammalian α-glucosidases and, thus, the potential use of *M. incana* extract in the management of postprandial hyperglycemia in type-2 diabetes.

The aerial parts of *Matthiola incana* represent an interesting source of bioactive compounds with antioxidant properties and the potential capability of preventing neurodegenerative or metabolic diseases through enzyme inhibition mechanisms. However, other investigations need to be carried out in living organisms in order to explore the bioactivities highlighted in this study and to identify the active compounds.
